# Yeast as a tool to explore cathepsin D function

**DOI:** 10.15698/mic2015.07.212

**Published:** 2015-07-11

**Authors:** H. Pereira, C.S.F. Oliveira, L. Castro, A. Preto, S. R. Chaves, M. Côrte-Real

**Affiliations:** 1 CBMA- Centre of Molecular and Environmental Biology. Department of Biology, University of Minho, Campus de Gualtar, 4710-057, Braga, Portugal.; 2 ICBAS - Institute of Biomedical Sciences Abel Salazar, University of Porto, 4050-313, Porto, Portugal.

**Keywords:** cathepsin D, cancer, apoptosis, yeast cathepsin D, yeast model

## Abstract

Cathepsin D has garnered increased attention in recent years, mainly since it has been associated with several human pathologies. In particular, cathepsin D is often overexpressed and hypersecreted in cancer cells, implying it may constitute a therapeutic target. However, cathepsin D can have both anti- and pro-survival functions depending on its proteolytic activity, cellular context and stress stimulus. Therefore, a more detailed understanding of cathepsin D regulation and how to modulate its apoptotic functions is clearly needed. In this review, we provide an overview of the role of cathepsin D in physiological and pathological scenarios. We then focus on the opposing functions of cathepsin D in apoptosis, particularly relevant in cancer research. Emphasis is given to the role of the yeast protease Pep4p, the vacuolar counterpart of cathepsin D, in life and death. Finally, we discuss how insights from yeast cathepsin D and its role in regulated cell death can unveil novel functions of mammalian cathepsin D in apoptosis and cancer.

## CATHEPSINS

Cathepsins are members of a large protease family, which can be subdivided according to their structure and active-site amino acid into cysteine (cathepsins B, C, F, H, K, L, O, S, V, W, and X), serine (cathepsins A and G), and aspartic cathepsins (cathepsins D and E). While cathepsins B, L, H, C and D are ubiquitously expressed in human tissues, expression of cathepsins A, G, K, S, V, X and W is tissue and cell type specific 1,2,3,4. In general, cathepsins are found in acidic cellular organelles, lysosomes and endosomes. Initially, their function was thought to be limited to bulk degradation of proteins delivered to the lysosome by endocytosis or autophagocytosis. However, it was later demonstrated that cathepsins possess highly specific and directed proteolytic activity, and that they can be found in other cellular compartments 5,6,7,8,9,10. Numerous physiological functions of cathepsins have been uncovered, including a role in hormone and antigen processing, bone and tissue remodeling, growth factor and proenzyme activation and, more recently, in the immune response 5,6,11,12,13. Cathepsins also participate in apoptosis and are translocated from the lysosomal lumen to the cytosol of mammalian cells through lysosomal membrane permeabilization (LMP) in response to a variety of apoptotic signals 14,15,16. These lysosomal proteases can also be secreted from the cell and degrade extracellular matrix proteins such as collagen, fibronectin, proteoglycans and laminin 17.

In addition to their physiological function, cathepsins have also been associated with several pathologies such as cardiovascular diseases, osteoporosis, rheumatoid arthritis, atherosclerosis and cancer 6,11,17,18,19. Elucidating the mechanisms underlying the involvement of cathepsins in the pathogenesis of these diseases, and how they can be modulated to develop new prevention and therapeutic strategies, has therefore taken center stage. Among cathepsins, cathepsin D (CatD) has attracted increased attention in recent years due to its importance in the mediation of lysosomal cell death pathways and in cancer. In this review, we will concentrate on both physiological and pathological functions of CatD, as well as on yeast as a model system to study CatD pathophysiology.

## ROLE OF CATHEPSIN D IN CELLULAR PHYSIOLOGY AND PATHOLOGY

CatD is a soluble aspartic endopeptidase found in the lysosomes of most mammalian cells. Like other cathepsins, CatD is activated by proteolytic cleavage of the synthetized inactive zymogen (preproCatD), which is composed of an N-terminal signal peptide, a propeptide, and a catalytic domain 20,21,22. The signal peptide directs the nascent chain to the endoplasmic reticulum, where it is cleaved in the lumen. ProCatD is then N-glycosylated and transported to the Golgi, where the N-glycan structures acquire mannose-6phosphate (Man-6P) residues that can bind to Man-6P receptor(s) (Man-6PR), and the complex is directed to the lysosomal compartment 23. In the acidic milieu, proCatD (52 kDa) undergoes further proteolytic processing by cleavage of the proregion, resulting in the 48 kDa single chain intermediate active form. Finally, this chain is processed into mature active CatD, composed of heavy (34 kDa) and light (14 kDa) chains linked by non-covalent interactions 24,25,26. It has been shown that CatD processing involves cysteine cathepsins 26,27 and, more recently, that it is independent of its own catalytic function and auto-activation but requires CatL and CatB 28. Although proCatD and CatD are mostly intracellular, they can also localize in the extracellular matrix and synovial fluid of cartilage 29,30,31. ProCatD/CatD are also found in human, bovine and rat milk 32,33,34, serum, sweat and urine 35,36, and extracellularly in macrophage-rich regions of atherosclerotic lesions 37. ProCatD secretion by human keranocytes 38, mammalian epithelial cells 39 and different types of cancer cells 18,40 was also demonstrated.

It is widely accepted that the major function of CatD is its involvement in general protein degradation and turnover within the lysosomal compartment. However, CatD has also emerged as an important regulator and signaling molecule with numerous physiological functions. These include activation of enzymatic precursors, prohormones and growth factors, processing of brain-specific antigens, tissue homeostasis, and participation in apoptosis 18,41. CatD has also been associated with different pathological scenarios such as cancer progression and metastasis, Alzheimer’s disease, atherosclerosis and inflammatory disorders 11,12,40,42, and found to be a specific biomarker for several pathologies. The involvement of CatD in both physiological and pathological processes has been addressed in multiple studies, some of which are summarized in Table 1 38,43,44,45,46,47,48,49,50,51,52,53,54,55,56,57,58,59,60,61,62. A more detailed description of the role of CatD in cancer is given below.

**Table 1:** Cellular roles of cathepsin D in physiological and pathological processes.

**Role**	**Model**	**References**
Limited proteolysis of proteins regulating cell growth and/or tissue homeostasis	*In vivo*: CatD-deficient mice	43
Postnatal tissue homeostasis including tissue renewal, remodeling, aging and RCD	*In vivo*: CatD-deficient mice; CatD-mutant mice	44,45,246,47
Neuronal ceroid lipofuscinosis in both animals and humans characterized by severe neurodegeneration, developmental regression, visual loss and epilepsy	*In vivo*: CatD-deficient mice; CatD-mutant mice Human pathologies Animal diseases	45 48 49 50 51
Wound healing, epidermal differentiation and pathological conditions such as psoriasis	*In vivo:* CatD-deficient mice *In vitro*: normal and psoriatic keranocytes Human patients	52 53 54
Proliferation and regeneration in keratinocytes and possibly in skin regeneration	*In vitro:* keratinocyte cell line HaCaT	38
Processing of proteins involved in Alzheimer disease pathogenesis, such as apolipoprotein E (apoE) and Tau protein	Human patients Recombinant protein	55 56
Post-partum cardiomyopathy resulting in heart failure	*In vivo*: mutant mice	57
Autism pathogenesis	Autistic subjects	58
Innate immune responses and Parkinson disease	*In vivo*: CatD-deficient mice Human patients	59 60
Intracellular metabolism, transport of phospholipids and cholesterol	Human patients	61
Atherosclerotic lesions associated with proCatD release from monocyte-derived macrophages	Atherosclerosis patients *In vitro*: cultured atherosclerotic plaques	62

## THE ROLE OF CATHEPSIN D IN CANCER

Numerous reports have demonstrated that CatD is overexpressed in several cancer types 18,40,42,63,64,65, often correlating with poor prognosis. In particular, CatD is considered an independent prognostic marker in breast cancer associated with metastatic risk 66,67,68 and in colorectal cancer (CRC) 69,70. Mechanistically, the majority of reports attribute its role in cancer to overexpression of proCatD. As an example, transfection of rat tumor cells with human proCatD cDNA leads to increased proliferation, invasion and metastasis *in vitro* and *in vivo *71. Accordingly, anti-proCatD antibodies can inhibit tumor growth both *in vitro* and *in vivo *72,73,74. Overexpressed proCatD escapes normal targeting routes and is hypersecreted to the extracellular milieu, where it can act in multiple fashions. On one hand, it can exert an autocrine effect, inducing cancer cell growth by interacting with cell surface receptors 72,75,76,77. This autocrine role has so far been observed in breast, prostate, ovarian and lung cancer cells 72,73,74,78. In addition, proCatD can play a crucial paracrine role in the tumor microenvironment by stimulating fibroblast outgrowth and tumor angiogenesis 71,79, as well as inhibiting anti-tumor responses 80. When in the tumor microenvironment, proCatD may also affect stromal cell behavior and/or degrade components from the extracellular matrix 81,82, including the release of growth factors 83. Although it has been suggested that proCatD can be processed in the acidic extracellular space to catalytically active CatD 84, the enzymatic activity of CatD is reportedly not required for its mitogenic role. Indeed, a proteolytically inactive mutant of CatD (D231N) is still mitogenic for fibroblasts 85, as well as for cancer cells both *in vitro*, in three-dimensional matrices, and in athymic nude mice 71,86. Similarly, proCatD stimulates angiogenesis in tumor xenografts of athymic nude mice independently of its catalytic activity 85, also suggesting that CatD can signal through protein-protein interactions.

Though less extensive, there are also examples of CatD roles in cancer cells that are not attributed to proCatD. For instance, intracellular CatD can stimulate cancer cell growth by inactivating secreted growth inhibitors 87,88. Moreover, mature CatD released into the cytosol as a consequence of the reportedly higher susceptibility of cancer cells to LMP 15,89 may interact with and/or degrade pro- and anti-apoptotic proteins, modulating cell death 41.

Targeting CatD is a promising strategy in the clinic, but requires further detailed elucidation of its mechanisms of action. In the following section, we focus on the role of CatD in the apoptotic process, which is of particular relevance for cancer research. These studies may however also offer clues into the function of CatD in other physiological and pathological scenarios.

## OPPOSING FUNCTIONS OF CATHEPSIN D IN APOPTOSIS

In recent years, multiple studies have shown that CatD is a central player in the apoptotic response, both under physiological and pathological conditions. In fact, depending on the cell type and context, CatD can induce or inhibit apoptosis, acting through different mechanisms 41. On one hand, CatD can directly induce apoptosis triggered by several stimuli such as staurosporine 90, etoposide, 5-fluorouracil and cisplatin 91, as well as resveratrol 92 and others, possibly mediated by intrinsic or extrinsic pathways 41. In the intrinsic pathway, the role of CatD is linked to the release of mature 34 kDa CatD into the cytosol and cleavage of Bid to form tBid, triggering insertion of the pro-apoptotic protein Bax into the mitochondrial membrane 15. Subsequent mitochondrial outer membrane permeabilization leads to the release of pro-apoptotic molecules such as cytochrome *c *and apoptosis inducing factor (AIF) to the cytosol 15. For instance, it has been shown that CatD mediates cytochrome *c *release and caspase activation in human fibroblasts undergoing staurosporine-induced apoptosis 90, and cleaves Bid and promotes apoptosis via oxidative stress-induced LMP in human neutrophils 93. In addition, Pepstatin A and/or knockdown of CatD expression by RNA interference prevent resveratrol toxicity, impeding Bax oligomerization, mitochondrial membrane permeabilization, cytochrome *c* release and caspase 3 activation in DLD1 and HT29 CRC cell lines 92. One study also reports that CatD mediates selective release of AIF in T lymphocytes entering the apoptosis early commitment phase through activation of Bax in a Bid-independent manner 94. This shows that CatD can be involved in caspase-independent apoptosis by activating Bax independently of Bid cleavage. Other studies strongly suggest that cytosolic CatD may have an additional role involving protein-protein interactions. As examples, it has been shown that overexpression of either catalytically active or inactive CatD by cancer cells enhances apoptosis-dependent chemo-sensitivity 95, and that stress-induced apoptosis is not affected in fibroblasts synthesizing a catalytically inactive CatD 96. Additionally, microinjection of inactive proCatD into the cytosol of both human fibroblasts and HeLa cells induces apoptosis 97. Interestingly, one report also indicates that cytosolic mature CatD may reach the nucleus during cell death 98.

In contrast with the multiple studies showing CatD is pro-apoptotic, other studies describe an anti-apoptotic function of CatD. Most of these suggest it plays an anti-apoptotic role in cancer cells. For example, CatD downregulation sensitizes human neuroblastoma cells to doxorubicin-induced apoptosis, while CatD overexpression has the opposite effect 99. Accordingly, inhibition of CatD with pepstatin A induces caspase-dependent apoptosis in neuroblastoma cell lines 100. Moreover, overexpression of intracellular CatD in mouse xenografs using rat-derived cell lines inhibits apoptosis 71, and expression of wild type or a catalytic mutant of CatD promotes survival and invasive growth of CatD‐deficient fibroblasts 85. Another study in glioblastoma cells proposes that CatD stimulates autophagy induction, inhibiting apoptotic cell death under genotoxic conditions 101. More recently, we showed that inhibition of CatD in CRC cells with small interfering RNA (siRNA) or pepstatin A enhances acetate-induced apoptosis associated with a decrease in mitochondria degradation independently of autophagy 102,103. An anti-apoptotic role of CatD has also been described under physiological conditions using CatD-deficient mice 43,44,45. Indeed, mutant mice developed apoptosis in the thymus, thalamus and retina.

In summary, it is well documented that CatD plays an important role in apoptosis regulation, both with and without involvement of its proteolytic activity. However, the exact role of CatD in apoptosis, particularly what determines whether this protease plays an anti- or pro-apoptotic function remains poorly understood. In this regard, a simpler model system would be particularly useful to offer additional clues into this dichotomy.

## YEAST VACUOLAR PROTEASES

The versatility of the yeast *Saccharomyces cerevisiae* to study several conserved cellular functions such as cell metabolism, cell cycle, cell death and organelle biogenesis has justified the attractiveness of this system to study more complex mammalian physiological and pathological processes 104,105,106,107,108. Like other organelles, the yeast vacuole is functionally similar to its higher eukaryote counterpart, the lysosome. It harbors seven characterized proteases, namely three aminopeptidases, three serine proteases and one aspartyl protease. Among these, two are endopeptidases: proteinase A (Pep4p), ortholog to human CatD, and proteinase B (Prb1p). Five are exopeptidases: carboxypeptidase Y (CPY), ortholog to human CatA, carboxypeptidase S (CPS1), aminopeptidase I (Ape1) and Y (Ape3), and dipeptidylaminopeptidase B (Dap2).

More recently, Hecht *et al.* reported an eighth vacuolar protease, a transmembrane metalloprotease (Pff1) 109, but although evidence of Pff1 vacuolar localization was shown, its proteolytic activity has yet to be demonstrated.

The endopeptidases are responsible for the majority of bulk protein degradation, including of plasma membrane proteins. They are also fundamental for activation of the vacuolar proteolytic cascade, particularly Pep4p, since it is involved in proteolytic activation of Prb1p, CPY and Ape1 110,111. Prb1p, in turn, participates in the activation of Pep4p, CPY, CPS1, Ape1 and Ape3. Both carboxypeptidases and Ape1 are involved in peptide and glutathione degradation, respectively, but are not required for zymogen activation 111,112.

Substrates for the vacuolar proteases are mostly imported via endocytosis (extracellular and cell surface proteins) or autophagy (cytoplasmic material and organelles). Autophagy is activated under nutrient deprivation conditions, and both Pep4p and Prb1p are implicated in the dissolution of autophagic bodies 113,114.

In addition, vacuolar proteases play a role in sporulation. While absence of Prb1p activity alone results in partial reduction of sporulation, absence of Prb1p activity in a mutant lacking both CPY and CPS1 leads to almost complete loss of sporulation ability 115. In addition to ensuring protein homeostasis under physiological conditions, vacuolar proteolysis therefore also appears to be a stress-responsive process, particularly under nutrient stress conditions and during sporulation. However, additional roles for vacuolar proteases have emerged in recent years, in particular for Pep4p.

## Pep4p PROTEASE - THE YEAST CATHEPSIN D

Yeast CatD (Pep4p), like its lysosomal counterpart, is synthesized as an inactive zymogen, traveling via the endoplasmic reticulum and Golgi to the acidic vacuoles, where it is activated through proteolytic removal of the inhibitory propeptide 116. Although Pep4p is mainly located in the vacuole, different cell death stimuli can lead to its release to the cytosol, involving a selective vacuolar membrane permeabilization (VMP) typical of apoptotic death.

Mason *et al*. were the first to report that Pep4p translocates from the vacuole to the cytosol 117. These authors observed an increase in nuclear permeability associated with increased accumulation of reactive oxygen species (ROS) during H_2_O_2_-induced cell death, and found that Pep4p is released into the cytosol and degrades nucleoporins during this process. However, Pep4p did not affect resistance to H_2_O_2_-induced cell death, probably because it migrates out of vacuoles after cells are effectively unviable. They further showed that the release of a Pep4p-EGFP (Enhanced Green Fluorescent Protein) fusion from the vacuole in H_2_O_2_-treated cells was not associated with major rupture of the vacuolar membrane, as cells maintained a vacuolar lumen morphologically distinct from the cytosol. Other authors reported that Pep4p is involved in protein degradation and removal of oxidized proteins during H_2_O_2_-induced oxidative stress, but also did not ascribe a role for this protease in cell death induced by H_2_O_2 _118.

Another study showed that stabilization of the actin cytoskeleton caused by lack of the actin regulatory protein End3p leads to loss of mitochondrial membrane potential, accumulation of ROS, increase in VMP and consequent migration of Pep4p to the cytosol, as well as apoptotic cell death 119. In that study, Pep4p-EGFP was visualized exclusively in the vacuole lumen in wild type cells, but distributed throughout the entire cell in an *END3*-deficient strain. Again, no role was attributed to this protease in actin-stabilized dying cells.

Pep4p is also involved in programmed nuclear destruction during yeast gametogenesis 120. Using cells co-expressing Pep4p-mCherry and Vma1-GFP, a GFP-tagged vacuolar membrane protein, Pep4p was shown to translocate from the vacuole into the ascal compartment of early postmeiotic cells during sporulation, with preservation of vacuolar integrity.

These observations show that VMP seems to mimic LMP in human cells. However, they do not indicate whether yeast vacuolar proteases play a role in cell survival and regulated death.

In this regard, it has been shown that Pep4p has a pro-survival role during chronological aging, since a Pep4p-deficient mutant has a shortened lifespan associated with higher levels of carbonylated proteins 118. Carmona-Gutiérrez *et al*. further showed that deletion of *PEP4* results in both apoptotic and necrotic cell death during chronological aging 121. Using a panel of Pep4p mutants, they conclude that Pep4p plays a dual pro-survival role composed of both anti-apoptotic and anti-necrotic functions, conferred by its proteolytic activity and its proteolytically inactive propeptide, respectively. We also previously found that Pep4p-EGFP translocates to the cytosol during acetic acid-induced apoptosis involving selective VMP in *S. cerevisiae *W303 cells, with preservation of both vacuolar and plasma membrane integrity 122. Moreover, we demonstrated that Pep4p is required for increased cell survival and for efficient autophagy-independent mitochondrial degradation in response to this acid in a manner depending on its catalytic activity 122,123. This suggests that VMP associated with Pep4p release may act as an alternative mitochondrial degradation process, delaying cell death. In contrast, we recently demonstrated that absence of *PEP4* resulted in increased resistance to acetic acid in *S. cerevisiae *BY4741 cells 124. This prompted the hypothesis that Pep4p plays a dual function in acetic acid-induced cell death depending on the genetic background, providing an interesting tool to explore the molecular determinants of CatD function.

## YEAST AS A TOOL TO EXPLORE THE ROLE OF CATHEPSIN D IN APOPTOSIS AND CANCER

It is widely established that the process of regulated cell death (RCD) involves a genetically encoded molecular machinery 125. Core components of this machinery are conserved in yeast, which can undergo RCD exhibiting typical markers of apoptosis, autophagy and necrosis 126,127,128. Thus, this eukaryotic organism has been used extensively to study the molecular mechanisms of RCD pathways, reviewed elsewhere 126,127,128,129. These studies encompass not only analysis of yeast endogenous death pathways but also heterologous expression of human proteins involved in apoptosis, such as caspases, Bcl-2 family proteins, PKC isoforms and the p53 tumor suppressor protein 130,131.

As discussed above, the role of the lysosome-like vacuole in the regulation of RCD has been investigated in yeast, where it has been shown to play a role similar to lysosomes 132,133. However, the use of this model organism to study lysosomal cell death pathways in general and cathepsin function in particular is still underexplored. So far, only translocation of Pep4p to the cytosol during yeast apoptosis has been clearly demonstrated by different authors 117,119,122. One other study shows that the RNase T2 family member Rny1p is also released from the vacuole into the cytosol during oxidative stress, with preservation of vacuolar membrane integrity, directly promoting cell death 134. The need for a comprehensive analysis of the VMP process and the vacuolar proteins released in response to different stimuli is therefore evident.

Another approach that has not been sufficiently exploited is the heterologous expression of cathepsins in yeast. Two studies have shown that rat cathepsin L and D precursor polypeptides are recognized by mechanisms similar to those involved in the intracellular sorting of vacuolar proteins in yeast cells 135,136. We therefore sought to further explore this tool to understand the function of human CatD. As mentioned above, we previously showed the parallel between the role of human and yeast CatD in acetate/acetic acid-induced apoptosis and in the degradation of damaged mitochondria, which render CRC/yeast cells more resistant to apoptosis induced by acetate/acetic acid 102,122. We now found that heterologous expression of human CatD in yeast *PEP4*-deficient cells reverts their sensitivity to acetic acid-induced apoptosis and delays mitochondrial degradation 103, as previously observed for wild type Pep4p 122,123. These results provide evidence that the role of CatD in both apoptosis and mitochondrial degradation is conserved through evolution. Further elucidation of the molecular mechanisms underlying the involvement of CatD in apoptosis and in mitochondrial degradation will now be crucial to develop novel strategies to specifically inhibit this protease in apoptosis deficiency-associated diseases, such as cancer.

**Figure 1:**
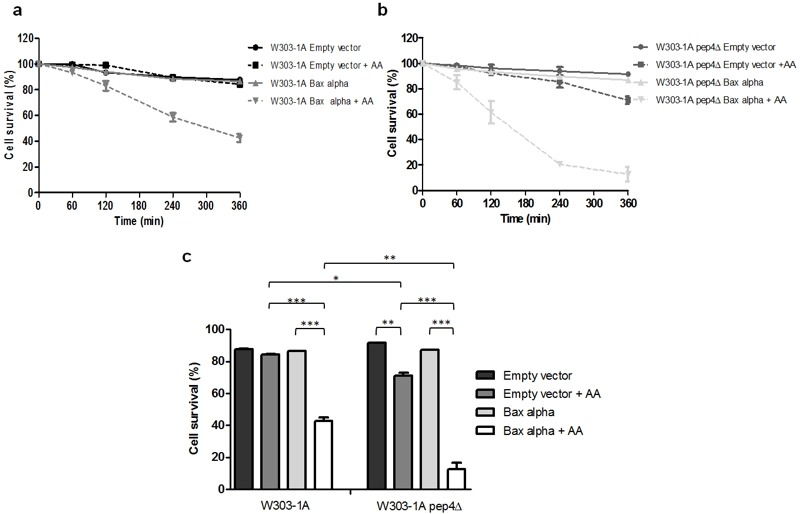
FIGURE 1: Survival of S. cerevisiae cells expressing Bax during acetic acid treatment. The wild type W303-1A and pep4Δ mutant strains transformed with the empty vector (PYES2) and PYES2-Bax alpha were incubated with 120 mM acetic acid for up to 360 min. **(A) ** Cell survival of W303-1A strain and **(B) ** W303-1A pep4Δ strain for up to 360 min was determined by standard dilution plate counts and expressed as a percentage of c.f.u. in relation to time 0. Data represents means ± S.D. (n=2). **(C) ** Cell survival at time 360 min was determined by standard dilution plate counts and expressed as a percentage of c.f.u. in relation to time 0. Data represents means ± S.D. (n=2). *P < 0.05, **P < 0.01, ***P < 0.001.

Taking into account the multiple functions of CatD, one caveat of using CatD inhibitors could be a negative effect on Bax activation, release of cytochrome *c* and downstream caspase activation. To address this question, we exploited the well-established system of heterologous expression of Bax in yeast, which lacks obvious orthologs of the Bcl-2 family, and allows studying how absence of yeast CatD affects Bax activity without interference from other Bcl-2 family members. Using yeast cells heterologously expressing a cytosolic inactive form of human Bax, which was activated by exposure to acetic acid, we could discard this hypothesis since absence of Pep4p enhanced Bax-induced cell death (Figure 1). It will be interesting to further exploit this system with heterologous co-expression of Bax and human CatD, in order to dissect the role of this lysosomal protease in the regulation of Bax activity independently of Bid.

As a final conclusion, it becomes apparent that the approaches with yeast have already provided and can further offer new perspectives for an increased understanding of the role of CatD in mammalian apoptosis, and its implications in cancer. Indeed, studies with yeast further reinforce the use of this eukaryotic organism as a valuable model to identify and characterize novel RCD processes, and open the door to new clinical opportunities, with a substantial impact in public health.
